# Preclinical Evaluation of the Stability, Safety, and Efficacy of CD101, a Novel Echinocandin

**DOI:** 10.1128/AAC.00701-16

**Published:** 2016-10-21

**Authors:** Voon Ong, Grayson Hough, Michael Schlosser, Ken Bartizal, James M. Balkovec, Kenneth D. James, B. Radha Krishnan

**Affiliations:** a Cidara Therapeutics, Inc., San Diego, California, USA; b Seachaid Pharmaceuticals, Durham, North Carolina, USA

## Abstract

Fungal infections pose a significant public health burden with high morbidity and mortality. CD101 is a novel echinocandin under development for the treatment and prevention of systemic Candida infections. Preclinical studies were conducted to evaluate the metabolic stability, plasma protein binding, pharmacokinetics, toxicity, and efficacy of CD101 at various dose levels. CD101 was stable to biotransformation in rat, monkey, and human liver microsomes and rat, monkey, dog, and human hepatocytes. *In vitro* studies suggest minimal interaction with recombinant cytochrome P450 enzymes (50% inhibitory concentrations [IC_50_s] of >10 μM). Similar to anidulafungin, CD101 bound avidly (>98%) to human, mouse, rat, and primate plasma proteins. In a 2-week repeat-dose comparison study, CD101 was well tolerated in rats (no effects on body weight, hematology, coagulation, or urinalysis). In contrast, administration of anidulafungin (at comparable exposure levels) resulted in reduced body weight, decreases in red blood cell, hemoglobin, hematocrit, mean cell volume, mean corpuscular hemoglobin, platelet, and reticulocyte counts, increases in neutrophil and eosinophil counts, polychromasia, and decreased activated partial thromboplastin time. Elevated plasma transaminases, total bilirubin, cholesterol, and globulin, dark and enlarged spleens, and single-cell hepatocyte necrosis were also observed for anidulafungin but not CD101. Hepatotoxicity may be due to the inherent chemical lability of anidulafungin generating potentially reactive intermediates. A glutathione trapping experiment confirmed the formation of a reactive species from anidulafungin, whereas CD101 did not exhibit instability or reactive intermediates. CD101 showed antifungal activity against Candida and Aspergillus infections in neutropenic mice. These preclinical studies demonstrated that CD101 is chemically and metabolically stable, well tolerated with no hepatotoxicity, and efficacious as an antifungal agent.

## INTRODUCTION

Serious fungal infections pose a significant public health burden with high morbidity and mortality. Candidemia, in particular, carries a 35% mortality rate in the first 12 weeks after diagnosis (1). Since 2009, echinocandins have been increasingly recommended as a first-line treatment for invasive fungal infections, such as candidemia, and as empirical therapy for suspected candidiasis in both neutropenic and nonneutropenic patients (2, 3). Echinocandins prevent fungal growth through inhibition of glucan synthase, which catalyzes synthesis of 1,3-β-d-glucan, a major component of cell walls in fungi but not mammals (4–6). Three echinocandins—caspofungin, micafungin, and anidulafungin—are currently approved for the once-daily treatment of candidemia and various manifestations of invasive candidiasis by the U.S. Food and Drug Administration.

Polyenes and azoles have also demonstrated efficacy in the treatment of systemic fungal infections. However, associated adverse effects and drug interactions, such as nephrotoxicity following amphotericin B treatment and cytochrome P450 (CYP450) enzyme inhibition by azoles, preclude their use in many clinical situations. Caspofungin, micafungin, and anidulafungin are associated with far fewer adverse effects and drug interactions, yet there is room for improvement in these currently available echinocandins. All have demonstrated liver function test abnormalities and hepatotoxicity (5–7), and all undergo nonenzymatic chemical degradation to a ring-opened product under neutral to basic pH conditions (6, 8, 9). Caspofungin, in particular, has been shown to generate two potentially reactive intermediates following spontaneous chemical degradation (5).

CD101, a novel echinocandin being developed as a high-exposure, once-weekly intravenous (i.v.) formulation for the treatment and prevention of systemic fungal infections, was designed to be chemically and metabolically stable to avoid hepatotoxicity while retaining the antifungal activity of an echinocandin. CD101 has demonstrated excellent stability in human plasma, with 93% remaining after 44 h, compared with 7% remaining for anidulafungin (10).

The preclinical studies described herein were conducted to evaluate the metabolic stability and pharmacokinetics of CD101 and to compare the antifungal effects and toxicities of CD101 and anidulafungin. In particular, the plasma protein binding characteristics, reactive metabolite profile, and hepatotoxicity of each antifungal agent were assessed at comparable exposure levels. Our findings support the further development of CD101 as a treatment of invasive fungal infections.

## MATERIALS AND METHODS

All studies of CD101 (Cidara Therapeutics, Inc., San Diego, CA) that involved animals received approval from an Institutional Animal Care and Use Committee, and all work was performed with appropriate local health regulations and ethical approval. Unless otherwise noted, stock solutions for *in vitro* studies were prepared in dimethyl sulfoxide (DMSO) and subsequently diluted into the appropriate buffer/medium for each experiment.

### Antifungal susceptibility testing.

MIC and minimal effective concentration (MEC) assays were performed according to the Clinical and Laboratory Standards Institute (CLSI) broth microdilution guidelines (11–13), with the exceptions that test compounds were made up at 50× the final assay concentration (100% DMSO) and 100-μl assay volumes were used. MIC and MEC assays were read following 24 h of incubation at 35°C for all drugs, except for amphotericin B, which was read at 48 h. MIC values for C. albicans were reported as the lowest concentration resulting in prominent growth inhibition (∼50%). MEC assays were read with the aid of a microscope, and values were reported as the lowest drug concentration at which rounded/compact hyphal morphology was observed. Enumeration of MIC and MEC assay inoculum viable counts was performed by plating aliquots of the starting assay inoculum on Sabouraud dextrose agar.

### Metabolic stability.

Metabolic stability was determined as described in reference 14. CD101 (1 μM) was incubated with liver microsomes and hepatocytes from Sprague-Dawley rats, cynomolgus monkeys, and humans or hepatocytes only from dogs with appropriate cofactors for up to 2 h at 37°C. Following incubation, samples were quenched with ice-cold methanol or acetonitrile (ACN), containing an appropriate internal standard and centrifuged to remove precipitated protein, and the supernatants were analyzed to quantitate the remaining parent. Samples from *in vitro* experiments were analyzed either by liquid chromatography-tandem mass spectrometry (LC-MS/MS) using an Agilent 6410 mass spectrometer coupled with an Agilent 1200 high-performance liquid chromatography (HPLC) device and a CTC PAL autosampler (microsomes) or an Applied Biosystems API4000 mass spectrometer coupled with a Shimadzu LC10 AD HPLC and a CTC PAL autosampler (hepatocytes). Two control agents (warfarin and verapamil) were analyzed under similar conditions. Data were converted to the percentage remaining by dividing by the time zero concentration value. Data were fit to a first-order decay model to determine half-life. Intrinsic clearance was calculated from the half-life and the protein concentrations.

### Plasma protein binding.

Ultracentrifugation (15) was used to compare human plasma protein binding of CD101 and anidulafungin. Plasma samples containing CD101, anidulafungin, or warfarin (1 and 5 μg/ml) were centrifuged at 99,000 rpm (∼500,000 × *g*) for 2.5 h at 37°C. Following ultracentrifugation, the supernatant was transferred to a microtiter plate containing 50 μl plasma for matrix-matching purposes and quenched with 400 μl of cold acetonitrile (ACN) with 0.1% formic acid containing diclofenac as an internal standard. Quenched samples were centrifuged for 5 min at 10°C (e.g., 3,100 rpm [2,000 × *g*]) to sediment the precipitated proteins, and 50 μl supernatant was transferred to a microtiter plate containing 100 μl of water prior to analysis by LC-MS/MS. The percentage bound was the difference between 100% and the percentage free (the ratio of the concentration in supernatant to the total concentration).

Rapid equilibrium dialysis (RED) (16) was used to determine cross-species protein binding of CD101 in CD-1 mouse, Sprague-Dawley rat, cynomolgus monkey, chimpanzee, and human plasma. CD101 (1 μM)- or warfarin (1 μg/ml)-treated samples were loaded into the plasma (right) channel of a RED device. Dialysis buffer (350 μl of phosphate-buffered saline [PBS], pH 7.4, with 100 mM sodium phosphate) was loaded into the buffer (left) channel of the RED device. The device was agitated gently at 37°C for 4 h. Samples were placed into a new 96-well plate for matrix matching (25 μl of plasma added to the dialysis buffer aliquots or 25 μl of dialysis buffer to the plasma aliquots). To each matrix-matched sample, 200 μl of 85.0 ng/ml combined d9-CD101 and d6-warfarin internal standard in 100% acetonitrile was added to precipitate the plasma proteins and provide internal standards. Following centrifugation, 200 μl of the supernatant was transferred to a clean 96-well plate and evaporated to dryness at 37°C under nitrogen. Samples were reconstituted in 100 μl of 1:1 acetonitrile-water prior to analysis by LC-MS/MS. The percentage bound was calculated as the difference between 100% and the percentage free (the ratio of the concentration in supernatant to the concentration in plasma). The percentage extraction recovery values were calculated based on the total (free and bound) concentration as a percentage of theoretical spiked concentration (1 μM CD101 and 1 μg/ml warfarin).

### CYP450 inhibition (17).

Serial dilutions of the CD101 stock solution (test concentrations of 0.03 to 30 μM) were prepared in a 9:1 solution of acetonitrile-dimethyl sulfoxide (DMSO) for CYP450 inhibition testing. The final DMSO content in the reaction mixture was equal in all solutions used within an assay and was less than 0.2%. The test articles were incubated at 7 increasing concentrations in duplicate with human liver microsomes in the presence of 2 mM NADP (NADPH) in 100 mM potassium phosphate (pH 7.4) containing 5 mM magnesium chloride and a probe substrate, in a 200-μl assay final volume. The probe substrates were amodiaquine (5 μM) for CYP2C8 and midazolam (2.5 μM) and testosterone (50 μM) for CYP3A4/5. Selective CYP inhibitors were screened alongside the test agents as a positive control, including quercetin (CYP2C8) and ketoconazole (CYP3A4/5). After incubation for 5 min at 37°C, the reactions were terminated by addition of methanol-containing internal standard (propranolol) for analytical quantification. The quenched samples were incubated at 4°C for 10 min and centrifuged at 4°C for 10 min. The supernatant was removed, and the probe substrate metabolite was analyzed by LC-MS/MS using an Agilent 6410 mass spectrometer coupled with an Agilent 1200 HPLC and a CTC PAL autosampler. A decrease in the formation of the metabolite compared to vehicle control was used to calculate a 50% inhibitory concentration (IC_50_ [the test concentration that produces 50% inhibition]).

### Dose range toxicity and hepatotoxicity screening.

In this repeat-dose study, CD101 (0, 2, 6, and 20 mg/kg body weight/day), anidulafungin (40 mg/kg body weight/day), and a vehicle control were administered as 20-min i.v. infusions over 14 days via lateral tail vein in 100 naive Sprague-Dawley rats (10 males and 10 females in each dose group, all ≥8 weeks old). Dosing solutions were prepared in a vehicle comprising 5% mannitol containing 2.5% polysorbate 80 (Tween 80) in 0.3% acetic acid.

Animals were randomly assigned to dose groups and stratified by weight to achieve similar group mean body weights (males, 255 to 318 g; females, 192 to 215 g). Blood, urine, and liver samples (excised and preserved in 10% neutral buffered formalin) were obtained on day 15. Plasma liver enzyme levels were assessed. Liver tissues were processed to paraffin blocks and prepared for slides. Slides were stained with hematoxylin and eosin. Hematology data were collected using the Advia 120 hematology analyzer. Coagulation data were collected using the Diagnostica STA compact coagulation analyzer. Clinical chemistry data were collected using the AU400 chemistry analyzer. Qualitative and quantitative urinalysis was recorded using iRICELL 1500 UA analyzer. Clinical effects were assessed for determination of the maximum tolerated dose (MTD).

Statistical evaluations were performed on in-life and clinical pathology numerical data to determine statistical significance following a decision tree. First, the homogeneity of the data was determined by Bartlett's test. If the data were homogeneous, a one-way analysis of variance was performed to assess statistical significance. If statistically significant differences between the means were found, Dunnett's test was used to determine the degree of significance from the control means (*P* < 0.05, *P* < 0.01, and *P* < 0.001). If the data were nonhomogeneous, the Kruskal-Wallis nonparametric analysis was performed to assess statistical significance. If statistically significant differences between the means were found (*P* < 0.05, *P* < 0.01, and *P* < 0.001), the Mann-Whitney U test was used to determine the degree of significance from the control means (*P* < 0.05, *P* < 0.01, and *P* < 0.001).

### Reactive metabolite screening.

For reactive metabolite screening (18), CD101 or anidulafungin was incubated in phosphate-buffered saline (PBS [pH 7.4]) alone and in the presence of a 5 M excess of reduced l-glutathione (GSH) at 37°C for 2 h. Samples were diluted to 10 μg/ml into 50:50 acetonitrile-water with 0.1 formic acid. Samples were analyzed by LC (Agilent 1100) with high-resolution MS (Waters quadrupole time of flight [Q-TOF] Premier mass spectrometer with electrospray interface) with concurrent low- and high-energy scans to obtain full-scan parent and product fragment ions. The analytical column was a Phenomenex Kinetex (2.6-μm C_18_, 100 by 3.0 mm). Waters MassLynx 4.1 software was used for data analysis.

### Preclinical efficacy. (i) Candida albicans.

Neutropenic male mice (20 to 24 g; *n* = 5/group) from the Institute for Cancer Research (ICR) were inoculated with C. albicans R303 (a human blood isolate from G. Denys at the Indiana University School of Medicine pathology lab) at 1.79 × 10^3^ CFU per mouse (0.2 ml/animal i.v.). Immunosuppression was induced by two intraperitoneal (i.p.) injections of cyclophosphamide at 150 mg/kg 4 days before and 100 mg/kg 1 day before inoculation. CD101 (MIC, 0.12 μg/ml) at 0.2, 0.4, 0.6 and 0.8 mg/kg, anidulafungin (MIC, 0.03 μg/ml) at 0.6 mg/kg, and vehicle (10% DMSO with 1% Tween 20 in saline) were administered once by i.v. injection 2 h after C. albicans infection. The reference substance, fluconazole (MIC, 2 μg/ml), was administered at 20 mg/kg by oral gavage 2 h after infection. The animals in the anidulafungin and CD101 groups were sacrificed, and the paired kidneys were harvested for colony count measurement (CFU per gram) 24, 48, or 72 h after inoculation. The animals in the fluconazole group were sacrificed 48 h after infection. A decrease of 99% or more (≥99%, 2 logs) in total CFU per gram of paired kidneys in the active groups compared to the vehicle group indicated significant antifungal efficacy.

### Preclinical efficacy (ii) Aspergillus fumigatus.

Neutropenic female mice (20 to 24 g; *n* = 10/group) from the ICR were inoculated with A. fumigatus (ATCC 13073) at 2.14 × 10^4^ CFU/mouse (0.2 ml/animal i.v.). Animals were immunosuppressed by three i.p. injections of cyclophosphamide (the first injection at 6 mg/mouse 3 days before inoculation and the second and third injections at 2 mg/mouse on 1 and 4 days after inoculation). CD101 (MEC, 0.004 μg/ml) at 0.2, 1, and 5 mg/kg i.v., anidulafungin (MEC, 0.004 μg/ml) at 1 and 5 mg/kg i.v., and vehicle (10% DMSO with 1% Tween 20 in saline) were administered twice daily (BID) for 5 days. Dose administration started 1 and 7 h after infection. The reference compound, amphotericin B (at 0.3 mg/kg; MIC, 0.125 μg/ml), was administered i.p. at 1 and 7 h postinfection and then twice daily for a total of 5 days. The mice were observed for mortality for 10 days. An increase of 50% or more in the survival rate of the active groups compared to the vehicle group indicated significant antifungal efficacy.

## RESULTS

### CD101 is stable to biotransformation.

**FIG 1:**
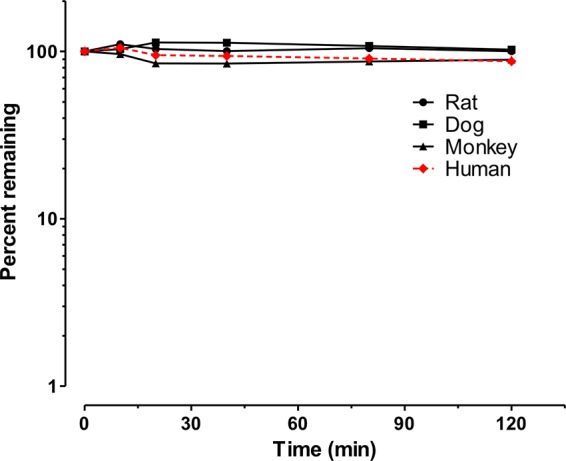
CD101 was stable when incubated across hepatocytes from different species.

During the *in vitro* study conducted to evaluate metabolic stability (clearance), CD101 was stable to biotransformation when incubated in liver microsomes and hepatocytes from humans, the Sprague-Dawley rat, and the cynomolgus monkey, as well as hepatocytes only from the dog. Approximately 100% of the CD101 remained after incubation with hepatocytes over the 2-h test period (Fig. 1). In liver microsomes, the half-life of CD101 was ≥240 min for rat, monkey, and human microsomes. Similarly, the half-life of warfarin, the stable control, was >240 min in all species. As expected, the half-life of verapamil, the metabolized control, was dependent upon NADPH: the half-life was >240 min in the NADPH-free environment but was substantially shorter in the presence of NADPH (Table 1). Microsomal intrinsic clearance of CD101 was 0 μl/min/ml in humans, regardless of NADPH. Comparable results were obtained in hepatocyte incubations. Positive controls, 7-ethoxycoumarin and 7-hydroxycoumarin for phase 1 and 2 biotransformations, respectively, confirmed the viability of the hepatocytes during the incubation.

**TABLE 1:** Microsomal intrinsic clearance and half-life of CD101, verapamil, and warfarin with and without NADPH in Sprague-Dawley rat, cynomolgus monkey, and human liver microsomes

Test agent^*a*^	Test species	NADPH dependent^*b*^		NADPH free^*b*^	
		Cl_int_ (μl/min/mg)	*t*_1/2_ (min)	CL_int_ (μl/min/mg)	*t*_1/2_ (min)
CD101 (1 μM)	Rat	9.6	239.6	0.0	>240
	Monkey	2.1	>240	0.0	>240
	Human	0.0	>240	0.0	>240
Verapamil (1 μM)	Rat	311	7.4	6.3	>240
	Monkey	890	2.6	3.1	>240
	Human	111	20.9	1.0	>240
Warfarin (1 μM)	Rat	7.3	>240	0.5	>240
	Monkey	6.8	>240	3.6	>240
	Human	3.2	>240	0.0	>240

a Verapamil served as the metabolized control and warfarin as the stable control.

b CL_int_, microsomal intrinsic clearance; *t*_1/2_, half-life.

### CD101 is highly bound to plasma proteins with values similar to those of anidulafungin.

The plasma protein binding levels of CD101 and anidulafungin were determined by two different methodologies: ultracentrifugation and equilibrium dialysis. The ultracentrifugation study compared the protein binding levels of CD101 and anidulafungin in human plasma and demonstrated that both compounds are highly protein bound (>98%), with no concentration dependence when tested at 1 and 5 μg/ml (Table 2). The equilibrium dialysis study demonstrated that CD101 is ≥98% protein bound, which is consistent with the results of the ultracentrifugation study. CD101 was highly protein bound in all animal and human plasma samples tested (99.1% in CD-1 mouse, 97.8% in Sprague-Dawley rat, 98.9% in cynomolgus monkey, 98.8% in chimpanzee, and 98.7% in human samples). As expected, warfarin was >98% bound in all species samples (99.5% in Sprague-Dawley rat, 99.3% in cynomolgus monkey, 99.5% in chimpanzee, and 98.1% in human samples), except for the mouse (91.8% in the CD-1 mouse sample).

**TABLE 2:** Human plasma protein binding of CD101 was comparable to that of anidulafungin, and CD101 shows the same protein binding values across various animal species

Agent	Concn (μg/ml)	% of protein bound (SEM)
CD101	1	98.0 (0.8)
	5	98.3 (0.5)
Anidulafungin	1	97.8 (0.8)
	5	98.6 (0.5)

### CD101 IC_50_s indicate minimal inhibition of CYP450 enzymes.

This study evaluated the impact of CD101 on the inhibition of recombinant CYP450 enzymes (1A2, 2B6, 2C8, 2C9, 2C19, 2D6, and 3A4). CD101 was further characterized using human liver microsomes to determine definitive IC_50_s for the CYP450-metabolizing enzyme 2C8 and 3A4 isoforms (Table 3). Consistent with other echinocandins, *in vitro* CYP inhibition studies suggest minimal interaction with CYP450 enzymes, with IC_50_s greater than 10 μM.

**TABLE 3:** Definitive IC_50_s of CD101 for the cytochrome P450-metabolizing enzyme 2C8 and 3A4 isoforms, characterized using human liver microsomes

Cytochrome P450 isoform	IC_50_ (μM)^*a*^
2C8 (amodiaquine substrate)	25.7
3A4 (midazolam substrate)	29.0
3A4 (testosterone substrate)	>30

a Molar mass = 1,226.39 g/mol; 1 μM = 1.23 μg/ml.

### No signs of hepatotoxicity were observed following CD101 administration in Sprague-Dawley rats.

In the 2-week, repeat-dose rat hepatotoxicity screening study, the incidence and severity of the clinical signs following CD101 administration were generally dose dependent, increasing from 2 mg/kg/day through 20 mg/kg/day. Clinical signs were primarily seen after the first two dose administrations and at 20 mg/kg/day were similar to those seen with anidulafungin. These transient clinical signs included bright extremities and/or ears, swollen ears and/or muzzle, red periorbital staining, clear ocular discharge, and an abnormal gait or stance. At comparable exposures to CD101 (20 mg/kg/day) and anidulafungin (40 mg/kg/day), additional transient clinical signs included mild to severe decreased activity, the animal feeling warm to the touch, labored respiration, bluish extremities, flaccid body tone, and vocalization. All animals, including vehicle controls, exhibited signs of tail vein irritation in the first few days after administration.

Animals who received CD101 did not exhibit effects on body weight, hematology, coagulation, or urinalysis, and there were no early deaths. In contrast, anidulafungin mean values for males and females relative to controls showed reduced body weights and food consumption (up to 9% decrease; *P* ≤ 0.05), decreases in red blood cells (RBC), hemoglobin, and hematocrit (up to 19% decrease; *P* ≤ 0.01), increases in RBC mean cell volume and mean corpuscular hemoglobin (up to 9% increase, *P* ≤ 0.001), increases in platelets (up to 44%; *P* ≤ 0.001) and in reticulocyte (polychromasia), neutrophil, and eosinophil counts (up to 4-fold; *P* ≤ 0.001), and decreases in activated partial thromboplastin time (up to 23% decrease; *P* ≤ 0.01). The majority of these changes were statistically significant compared with the corresponding values of the concurrent control group. Statistically significant increases for animals that received anidulafungin (relative to controls) were observed in mean alanine transaminase (ALT [up to 27%; *P* ≤ 0.05]), aspartate aminotransferase (AST [up to 68%; *P* ≤ 0.001]), alkaline phosphatase (ALP [up to 47%; *P* ≤ 0.001]), and total bilirubin (up to 2-fold; *P* ≤ 0.001). In addition, anidulafungin-treated rats displayed increases in serum cholesterol (up to 52%; *P* ≤ 0.01) and globulin (up to 19%; *P* ≤ 0.01), with an accompanying decrease in the albumin/globulin ratio (up to 15% decrease; *P* ≤ 0.001), as well as dark and enlarged spleens upon necropsy. The few statistically significant changes in animals that received CD101 were within normal limits for Sprague-Dawley rats of their age and were considered incidental and not related to CD101 administration; these consisted of increases in neutrophils (up to 31%; *P* ≤ 0.01) and increases in reticulocytes, hemoglobin, hematocrit, activated partial thromboplastin time, blood urea nitrogen (BUN), sodium, total bilirubin, total protein, chloride, and globulin (increases of less than 20%; *P* ≤ 0.05).

**FIG 2:**
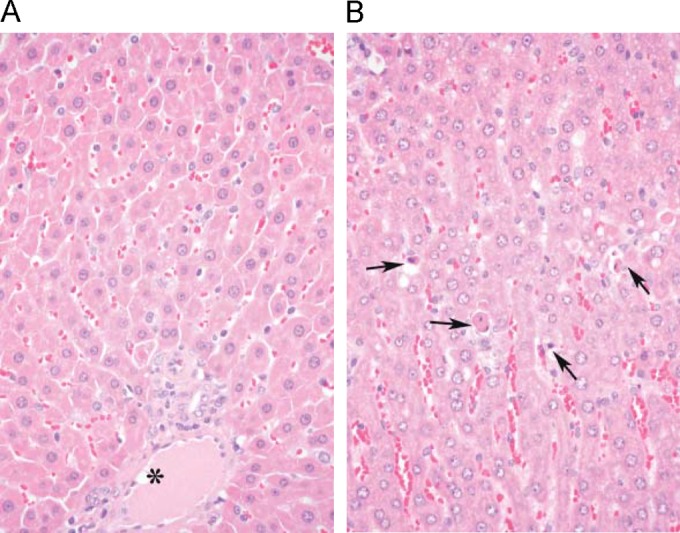
Comparative liver histopathology for (A) CD101 and (B) anidulafungin from the 2-week rat hepatotoxicity screening study. An asterisk shows the portal tract, while black arrows point to hepatocellular necrosis in the case of anidulafungin.

There were no microscopic changes in the livers of animals who received CD101 at 2, 6, or 20 mg/kg/day. With anidulafungin administration, minimal to moderate single-cell necrosis affecting the hepatocytes was observed in some of the females (Fig. 2). This finding was characterized by shrinkage of the hepatocyte, with increased eosinophilia of the cytoplasm and increased basophilia of the nuclei or their disintegration. Single-cell necrosis was considered related to administration of anidulafungin.

### No reactive intermediate was formed from CD101.

**FIG 3:**
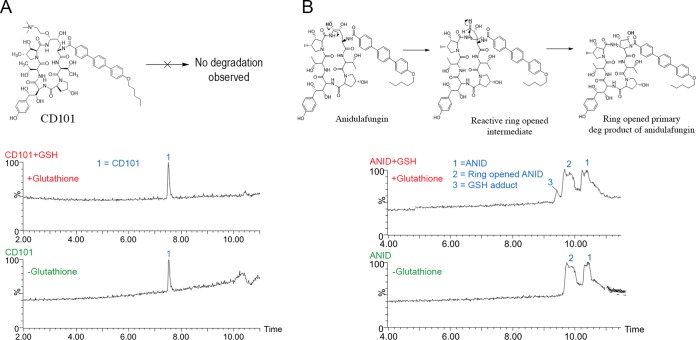
Following 24 h of incubation with and without GSH, (A) CD101 shows excellent stability, whereas (B) anidulafungin (ANID) shows evidence of reactive intermediate formation.

The reactive metabolic profiles of CD101 and anidulafungin were studied by LC with high-resolution MS detection following incubation with glutathione (GSH) in PBS at pH 7.4 and 37°C. CD101 was not found to generate any GSH adduct after incubation, indicating the absence of reactive intermediates (Fig. 3A). In contrast, anidulafungin did form a GSH adduct during incubation. Figure 3B shows evidence of reactive intermediate formation from the ring-opening degradation pathway of anidulafungin.

### CD101 demonstrated potent antifungal efficacy in a neutropenic mouse model of C. albicans infection.

**FIG 4:**
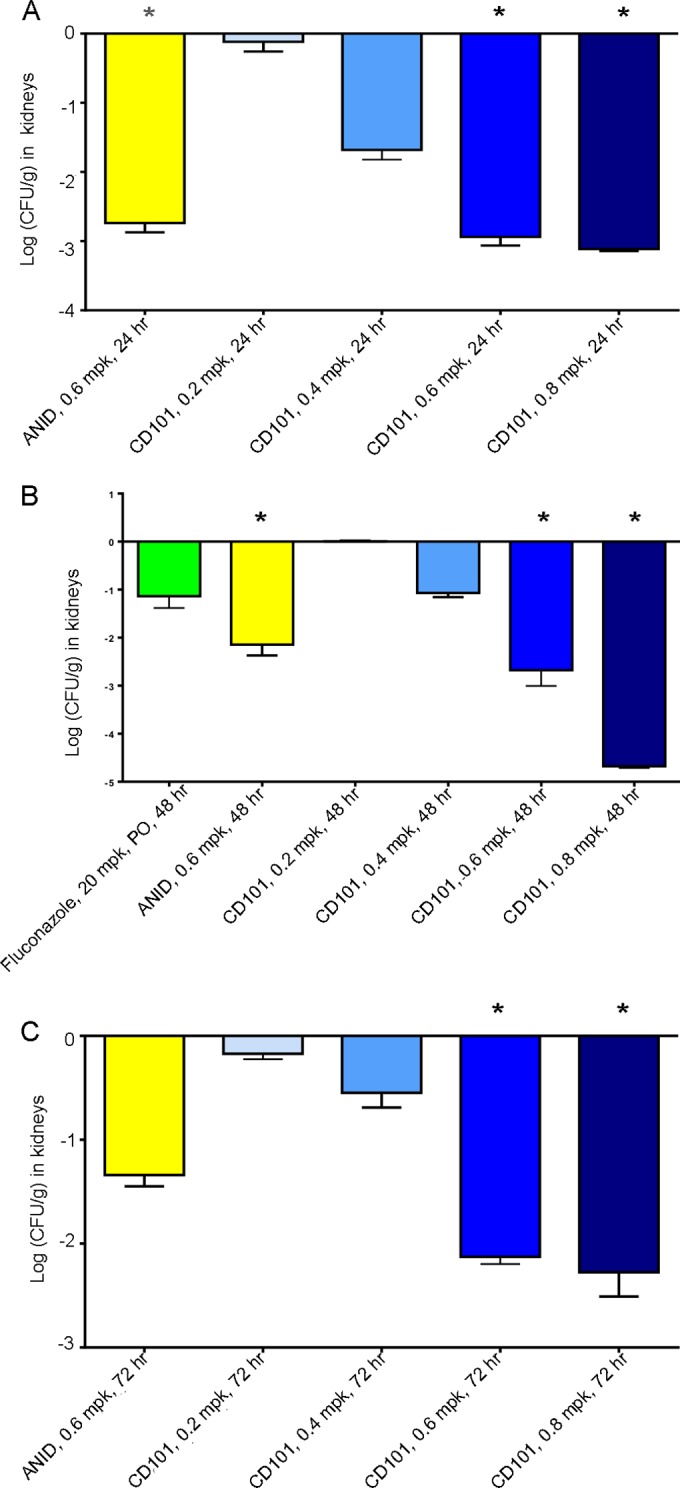
Reduction in Candida albicans colony counts (log CFU per gram) in kidney at (A) 24, (B) 48, and (C) 72 h following systemic (i.v.) infection and subsequent treatment with CD101, anidulafungin (ANID), and fluconazole (at 48 h only). Asterisks indicate a 2-log reduction (or more) relative to vehicle.

In the study of various doses of CD101 and anidulafungin in male neutropenic ICR mice inoculated with C. albicans, a dose response was seen following CD101 administration, with a 2% increase to a 41% decrease (less than 1-log reduction) in CFU per gram following the 0.2-mg/kg dose, 71 to 98% decreases (up to 1.7-log reduction) in CFU per gram following the 0.4-mg/kg dose, and 99 to ∼100% decreases in CFU per gram following both the 0.6-mg/kg (up to 2.9-log reduction) and 0.8-mg/kg (up to 4.7-log reduction) doses relative to mean CFU per gram in the vehicle group. Anidulafungin administration resulted in 96 to 100% decreases (up to 2.7-log reduction) in CFU per gram relative to mean CFU per gram in the vehicle-treated group. Fluconazole at 20 mg/kg (oral) elicited an 87% (or 1.1-log) reduction in CFU per gram relative to mean CFU per gram in the vehicle group. The antimicrobial effects were significant (≥99%, 2-log reduction in CFU per gram) in the groups receiving anidulafungin at 0.6 mg/kg (at 24 and 48 h) and CD101 at 0.6 and 0.8 mg/kg (at 24, 48, and 72 h) (Fig. 4). The 0.6- to 0.8-mg/kg doses of CD101 were the most effective of all the dosing groups tested against C. albicans in ICR mice.

### CD101 demonstrated potent antifungal efficacy in a neutropenic mouse model of A. fumigatus infection.

**FIG 5:**
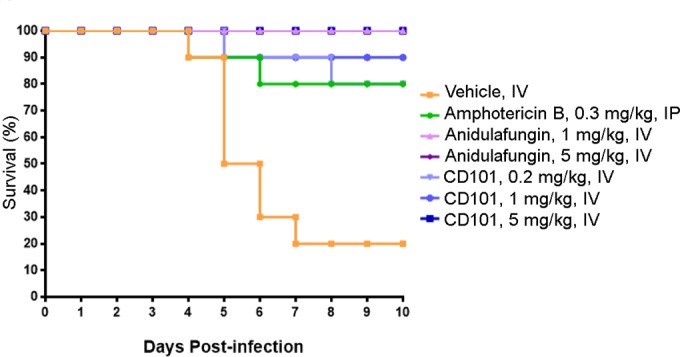
Survival of neutropenic ICR mice inoculated with Aspergillus fumigatus following administration of anidulafungin, CD101, amphotericin B, and vehicle BID for 5 days.

In the study of various doses of CD101 and anidulafungin in the A. fumigatus septicemia model, a dose response was observed following CD101 administration, with 80% survival following the 0.2-mg/kg dose, 90% survival following the 1-mg/kg dose, and 100% survival following the 5-mg/kg dose given BID for 5 days (Fig. 5). Both CD101 (all dose groups) and anidulafungin (1 and 5 mg/kg BID for 5 days) were associated with a significant increase in the 10-day survival rate compared to the vehicle group (*P* < 0.05). The reference compound, amphotericin B (0.3 mg/kg BID for 5 days), also elicited a significant increase in the survival rate compared to the vehicle group.

## DISCUSSION

CD101 is a novel echinocandin with exceptional pharmacokinetic and safety attributes that distinguish it as a next-generation echinocandin. CD101 was stable, with no evidence of biotransformation in liver microsomes and hepatocytes from humans and other animal species, as shown in the metabolic stability study (Fig. 1). Furthermore, no reactive intermediate formation was evident following incubation of CD101 with glutathione, in contrast to anidulafungin, which formed a reactive electrophilic species capable of covalently binding to nucleophilic sulfhydral groups under the same conditions (Fig. 3). Anidulafungin is known to undergo a nonenzymatic chemical degradation to a ring-opened product in the presence of neutral to basic pH conditions (6, 8, 9). Similarly, caspofungin has been shown to undergo spontaneous chemical degradation as well as generate two potentially reactive intermediates from this chemical degradation (5). The design of CD101 provides stabilization of the attached choline, preventing subsequent opening of the ring (10).

The stability of CD101 may partly explain why hepatotoxicity was not observed in rats following a 14-day, repeat-dose i.v. infusion of CD101. In this study, an increase in hepatocellular single-cell necrosis and levels of plasma liver enzymes were observed in animals that received anidulafungin. Single-cell hepatocellular necrosis, hepatocellular hypertrophy, and increased liver weight have been reported previously in animal studies with anidulafungin (6). Hepatotoxicity may be due to the inherent chemical lability of anidulafungin (or other currently available echinocandins) generating potentially reactive intermediates (8, 9). The lack of hepatotoxicity with CD101, compared with anidulafungin, in this study does not appear to be due to a difference in protein binding between the two compounds, as human plasma protein binding of CD101 was comparable to that of anidulafungin (Table 2).

In the 14-day, repeat-dose i.v. infusion study in rats, a similar set of transient, dose-dependent adverse clinical signs were observed with both CD101 and anidulafungin at comparable plasma exposure levels. Plasma exposure levels from the CD101 20 mg/kg and anidulafungin 40 mg/kg used in the toxicology comparison were considered to be comparable based on previously obtained pharmacokinetic data in the rat suggesting 1.5- to 3-fold-higher exposures from CD101 than from anidulafungin (normalized on a per-milligram-per-kilogram basis) (19; V. Ong, K. D. James, S. Smith, and B. R. Krishnan, submitted for publication). The clinical signs observed were indicative of a histamine release response, a well-characterized and species-specific effect seen with echinocandins in rats but not seen during regulatory toxicity studies in monkeys (5, 6). Rats appear to be much more sensitive to histamine release responses of echinocandins compared with primates, including humans, in whom histamine-mediated infusion reactions appear to be uncommon (5–7). Despite these transient adverse clinical signs, the CD101 dose of 20 mg/kg/day did not appear to elicit any evidence of repeat-dose systemic or target organ toxicity. In contrast, anidulafungin induced severe hemolytic anemia and liver toxicity, findings that had been previously reported for anidulafungin (20). Although it is unknown whether adverse effects reported with currently available echinocandins are due to the reactive intermediates or the parent compound, the formation of reactive intermediates has been associated with the toxicity of many compounds (21). The lack of such reactive intermediates and the safety of CD101 compared with anidulafungin at comparable exposures in these studies suggest a wider safety margin for CD101 and potential to safely achieve higher plasma drug exposures.

The safety profile of antifungal agents is of particular importance since candidemia is most common in adults aged ≥65 years or more, a population more likely to have comorbidities and be taking other medications (22, 23). Drug interactions between CD101 and drugs that are metabolized by CYP450 isoenzymes seem unlikely. The *in vitro* CYP450 inhibition study showed that CD101 does not appear to be a good substrate or inhibitor of CYP450 isoenzymes, which is consistent with the profiles of other echinocandins.

CD101 demonstrated potent antifungal efficacy against C. albicans and A. fumigatus in ICR mice. The response of neutropenic mice infected with A. fumigatus to CD101 administration was dose dependent, resulting in 80%, 90%, and 100% survival following the 0.2-, 1-, and 5-mg/kg doses. These results are consistent with the study of neutropenic mice infected with C. albicans, which showed up to 72 h of CD101 efficacy after a single dose. In this disseminated candidiasis study, in which only a single dose was administered, the prolonged efficacy may be the result of a longer plasma half-life of CD101 in comparison with other echinocandins. The difference in half-life is greater in primates than in mice (Ong et al., submitted), suggesting that prolonged efficacy may be extended in primates as well.

The studies described herein are part of a comprehensive series of safety, pharmacology, and toxicology studies of up to 4 weeks' duration as well as genetic toxicology studies, as required for an investigational new drug application. The results provide important insights for further research and development of CD101 and are best considered in this broader, developing context. In the present studies, preclinical efficacy was demonstrated against only single isolates of C. albicans and A. fumigatus. Additional, yet unpublished results showing comparable efficacy have been obtained with other C. albicans isolates. Although animal models of aspergillosis, such as the one used here and in similar studies (24), remain useful for drug evaluation and prediction of antimicrobial effect in humans (25, 26), the work toward improving future aspergillosis models (27, 28) and the challenges inherent in translational research in general are well recognized. The *in vivo* studies also demonstrated a long half-life and acceptable safety profile for CD101; the 4-week, repeat-dose studies in rats and cynomolgus monkeys established no observed adverse effect levels of 34 times and 47 times, respectively, the efficacious plasma exposure level as previously described (Ong et al., submitted). The pharmacokinetic/pharmacodynamic attributes of CD101 may allow for a more flexible dosing schedule than currently available echinocandins, and future clinical studies of CD101 are anticipated with once-weekly dosing based on phase 1 data in humans (T. Sandison, V. Ong, J. Lee, and D. Thye, submitted for publication).

In conclusion, the high stability, low clearance, long plasma half-life, and remarkable safety/efficacy profile of CD101 support further development of CD101 as a highly differentiated therapy for the treatment of serious fungal infections.
